# Translational Gap in Biomarker Discovery: Tumor Surface Markers Rarely Mirror Circulating Levels

**DOI:** 10.1002/ags3.70240

**Published:** 2026-06-05

**Authors:** Junko Mukohyama, Shohei F. Fujita, Xinyi Fu, Shigenori Suzuki, Hiroki Hamamoto, Dai Shida, Kimihiro Yamashita, Kohei Taniguchi, Sang‐Woong Lee, Akihide Yoshimi

**Affiliations:** ^1^ Division of Frontier Surgery, The Institute of Medical Science The University of Tokyo Tokyo Japan; ^2^ Department of Surgery, The Institute of Medical Science The University of Tokyo Tokyo Japan; ^3^ Division of Cancer RNA Research National Cancer Center Research Institute Tokyo Japan; ^4^ Department of General and Gastroenterological Surgery Osaka Medical and Pharmaceutical University Osaka Japan; ^5^ Division of Analytical Biomedical Sciences, Department of Biophysics Kobe University Graduate School of Health Sciences Kobe Japan; ^6^ Division of Translational Research Center for Medical Research & Development Osaka Medical and Pharmaceutical University Osaka Japan

**Keywords:** CDX2, cell surface proteins, circulating biomarkers, colorectal cancer, translational gap

## Abstract

**Background:**

Tumor‐associated cell surface proteins are frequently proposed as circulating biomarkers for colorectal cancer (CRC) based on their high tumor expression. However, many candidates identified through tissue‐based analyses fail to translate into clinically useful biomarkers. We investigated the translational gap between tissue‐level expression and circulating detectability in CRC, focusing on molecular subtypes defined by caudal‐type homeobox 2 (CDX2) expression.

**Methods:**

Transcriptomic data from The Cancer Genome Atlas (TCGA) were analyzed to identify cell surface markers differentially expressed between CDX2‐Low and CDX2‐High CRCs. A clinical cohort of right‐sided CRC patients was evaluated using paired tumor tissue and preoperative plasma samples. CDX2 expression was assessed by immunohistochemistry, and circulating concentrations of selected cell surface proteins were quantified using a multiplex ELISA platform.

**Results:**

Several tumor‐associated cell surface markers exhibited marked CDX2‐dependent differences in tissue expression. However, for most markers, circulating plasma levels did not mirror tissue‐level patterns. CEACAM1 was the sole marker demonstrating concordant CDX2‐dependent differences in both tumor tissue and plasma, with significantly lower levels in CDX2‐Low CRCs. In contrast, CEACAM5 showed a dissociation between tissue expression and circulating levels, despite analytical validation against serum carcinoembryonic antigen (CEA).

**Conclusions:**

Our findings demonstrate that tumor overexpression of cell surface markers does not necessarily translate into detectable circulating biomarkers. This translational disconnect underscores limitations of biomarker selection strategies based solely on tissue expression and highlights the importance of integrating systemic biology into biomarker development. While some tumor‐associated proteins may lack utility as circulating biomarkers, they may still represent viable therapeutic targets in CRC.

## Abstract 2

Tumor surface markers are attractive biomarker candidates, yet their circulating levels often fail to reflect tumor expression. Our findings in colorectal cancer reveal a marked tissue–plasma disconnect, underscoring a translational gap in biomarker discovery.

## 1 Introduction

Cell surface proteins highly expressed in tumor tissue are frequently proposed as biomarker candidates for cancer detection and/or monitoring. However, clinical applicability requires not only robust tissue expression but also reliable detectability and biological relevance in the circulation. In practice, many biomarkers identified through tissue‐based transcriptomic or proteomic analyses fail to translate into clinically useful blood‐based markers, highlighting a persistent gap between molecular discovery and clinical implementation.

CDX2 is an intestinal lineage transcription factor, stratifies colorectal cancer (CRC) into biologically distinct subtypes, with CDX2‐Low tumors exhibiting aggressive behavior and unique transcriptional profiles. Accumulating evidence indicates that reduced or absent CDX2 expression defines a biologically aggressive CRC subtype, characterized by poor differentiation, increased metastatic potential, and unfavorable prognosis [1, 2]. CDX2‐Low CRC exhibits molecular features associated with stemness, impaired differentiation, and dysregulated transcriptional programs, with experimental suppression of CDX2 inducing upregulation of microRNA‐221 and highlighting its role in regulating microRNA networks in CRC [3]. These findings suggest that CDX2 status provides a biologically meaningful framework for stratifying CRC and for exploring subtype‐specific biomarker candidates. Nevertheless, whether molecular features observed at the tissue level in CDX2‐defined CRC subtypes are recapitulated in the circulation remains largely unexplored. We investigated whether cell surface markers markedly overexpressed in CDX2‐Low CRC tissues are detectable in plasma and whether they reflect tissue expression patterns.

## 2 Methods

### 2.1 TCGA Data Analysis

We analyzed CRC transcriptomic data from The Cancer Genome Atlas (TCGA) database. RNA sequencing data and corresponding clinicopathological annotations were obtained from publicly available TCGA‐CRC datasets. Tumors were classified into CDX2‐Low and CDX2‐High groups based on CDX2 mRNA expression levels, using a predefined cutoff consistent with prior studies. Differential expression analysis was performed to identify cell surface–associated genes that were significantly differentially expressed between CDX2‐Low and CDX2‐High tumors. Statistical significance was determined after appropriate multiple‐testing correction. It should be noted that the TCGA transcriptomic dataset and the institutional plasma cohort were independent, and no direct tissue–plasma correlation analysis was performed within the same patients.

### 2.2 Clinical Cohort and Tissue Analysis

Resected primary CRC specimens were obtained from patients with right‐sided CRC who underwent surgical resection at Osaka Medical and Pharmaceutical University Hospital. From this surgical cohort, cases were selected in which both tumor tissue and preoperative plasma samples had been prospectively stored in the institutional biobank. All samples were collected in accordance with institutional ethical guidelines.

CDX2 expression in resected tumor specimens was evaluated by immunohistochemistry. Immunostaining was performed using an automated staining system with a mouse monoclonal anti‐CDX2 antibody (clone CDX2‐88; Abcam, Cambridge, UK) at a dilution of 1:100. Nuclear staining intensity and the proportion of CDX2‐positive tumor cells were independently assessed by experienced pathologists who were blinded to clinical information. Tumors were classified as CDX2‐Low or CDX2‐High according to established immunohistochemical criteria, as previously reported.

Preoperative peripheral blood samples were collected prior to surgical intervention. Plasma was isolated by centrifugation using standardized protocols and stored at −80°C until analysis. Only patients with paired tumor tissue and plasma samples available were included in the final analysis to ensure direct comparability between tissue‐ and circulation‐based assessments.

### 2.3 Multiplex ELISA Analysis

Plasma biomarker concentrations were quantified using a bead‐based multiplex immunoassay (Human Luminex Assay, R&D Systems) according to the manufacturer's instructions. The following cell surface–associated proteins were measured: CEACAM5 (CD66e), CEACAM1 (CD66a), ERBB2 (HER2), ERBB3 (HER3), and ALCAM (CD166). This platform enables simultaneous measurement of multiple analytes within a single plasma sample by using distinct sets of fluorescently labeled microspheres, each conjugated with a specific capture antibody. Following incubation with plasma samples, bound analytes were detected using biotinylated detection antibodies and streptavidin–phycoerythrin. Fluorescence intensity corresponding to each analyte was measured using a Luminex detection system, and concentrations were calculated from standard curves generated for each target protein according to the manufacturer's instructions. Assays were performed in duplicate, and concentration values were calculated from standard curves generated using recombinant proteins provided in the assay kits. Quality control procedures were applied to ensure assay reproducibility and reliability. Given the skewed distribution of circulating marker levels, data are summarized as median (IQR), while effect sizes are expressed as mean differences with 95% confidence intervals (Table S1).

### 2.4 Statistical Analysis

Associations between circulating protein levels and CDX2 status were evaluated using two‐way analysis of variance (ANOVA), followed by Bonferroni's multiple comparisons test where appropriate. Correlations between tissue‐level expression and circulating concentrations were assessed using correlation analyses. Clinicopathological variables, including tumor stage and nodal status, were compared between groups to evaluate potential confounding effects. A two‐sided *p*‐value < 0.05 was considered statistically significant. All statistical analyses were performed using Prism Version 8 (GraphPad Software, Boston, MA, USA).

## 3 Results

### 3.1 Differential Expression of Cell Surface Markers According to CDX2 Status in CRC Tissues

**FIGURE 1:**
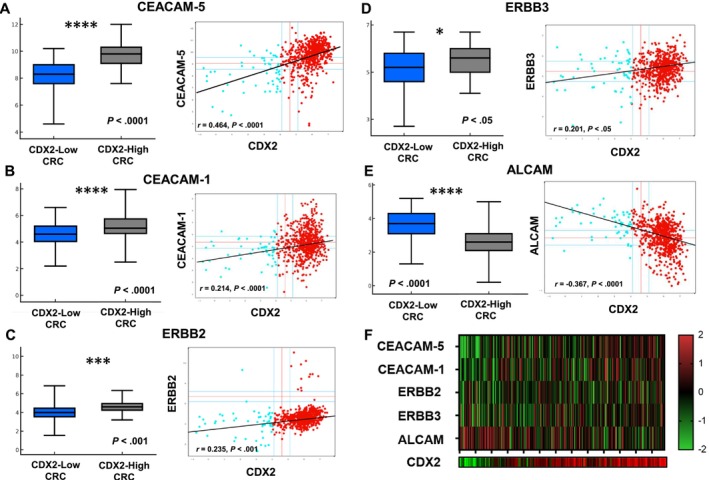
Differential expression of tumor‐associated cell surface markers according to CDX2 status in colorectal cancer (TCGA dataset). (A–E) Comparison of CEACAM5 (CD66e) (A), CEACAM1 (CD66a) (B), ERBB2 (HER2) (C), ERBB3 (HER3) (D), and ALCAM (E) gene expression between CDX2‐Low and CDX2‐High tumors based on The Cancer Genome Atlas (TCGA) colorectal cancer (CRC) dataset (left; box and bar represent the interquartile range and median, respectively; Whiskers 1.5 × IQR; one‐way ANOVA with Tukey's multiple comparisons test; *p < 0.05, ***p < 0.001, ****p < 0.0001). Correlation between CDX2 and the indicated gene is also shown (right; scatterplots showing correlations with CDX2 and least‐squares regression lines); Pearson's correlation analysis with *r* and *p*‐values shown. (F) Heatmap showing the expression of CDX2 and the five tumor‐associated cell surface markers based on TCGA colon adenocarcinomas.

Transcriptomic analysis of the TCGA CRC dataset demonstrated marked differences in the expression of multiple cell surface markers between CDX2‐Low and CDX2‐High tumors (Figure 1A–F). CEACAM5 and CEACAM1 expression levels were significantly reduced in CDX2‐Low CRCs compared with CDX2‐High CRCs (both *p* < 0.0001). In contrast, ALCAM expression was significantly higher in CDX2‐Low tumors (*p* < 0.0001). ERBB2 and ERBB3 expression levels were also modestly but significantly elevated in CDX2‐High tumors (*p* < 0.001 and *p* < 0.05, respectively). Correlation analyses across the TCGA cohort quantitatively assessed the relationship between CDX2 expression and selected cell surface markers. CDX2 expression showed a moderate positive correlation with CEACAM5 (*r* = 0.464, *p* < 0.0001) and an inverse correlation with ALCAM (*r* = −0.367, *p* < 0.0001), while the remaining markers exhibited weaker but statistically significant associations (Figure 1). In addition, heat map visualization of selected cell surface markers illustrated distinct expression patterns according to CDX2 status across the TCGA cohort (Figure 1F). This analysis highlights that CDX2‐Low and CDX2‐High tumors exhibit different overall expression profiles of tumor‐associated cell surface markers, without implying complete separation between the two groups. These data indicate that CDX2 status is closely associated with distinct tissue‐level expression patterns of several clinically relevant cell surface proteins.

### 3.2 Patient Selection and Classification Based on CDX2 Immunohistochemistry

**FIGURE 2:**
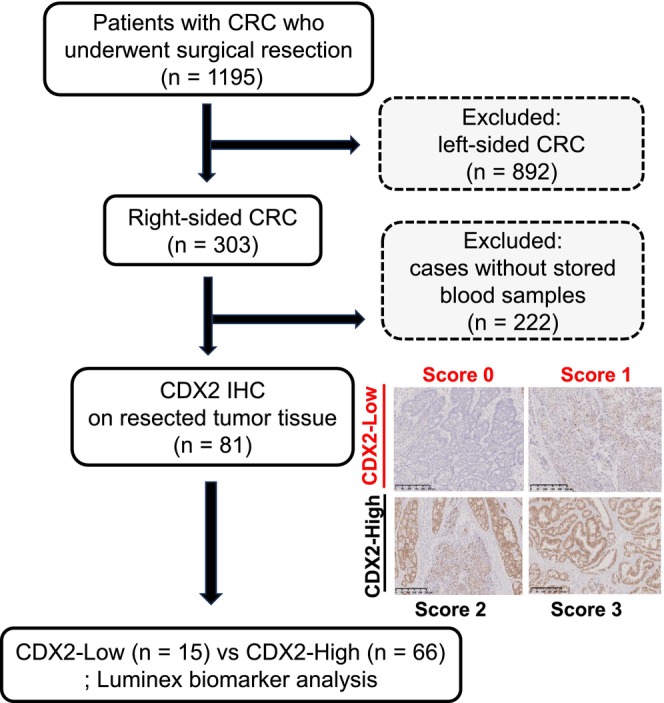
Study schema and CDX2 immunohistochemistry. Study schema. Resected CRCs were screened; left‐sided tumors and prespecified exclusions were removed. Eligible right‐sided cases with stored blood underwent CDX2 IHC and were grouped as CDX2‐Low (*n* = 15) or CDX2‐High (*n* = 66). Serum/plasma biomarkers were then measured by Human Luminex assay. CDX2 expression was assessed by immunohistochemistry based on nuclear staining intensity and classified into four categories: Score 0, no staining; Score 1, weak or scattered nuclear staining in a minority of tumor cells; Score 2, moderate to strong nuclear staining in a majority of tumor cells; and Score 3, strong nuclear staining in all tumor cells. CDX2‐Low was defined as Scores 0–1 and CDX2‐High as Scores 2–3. Representative images for each score are shown. Scale bar, 200 μm.

**FIGURE 3:**
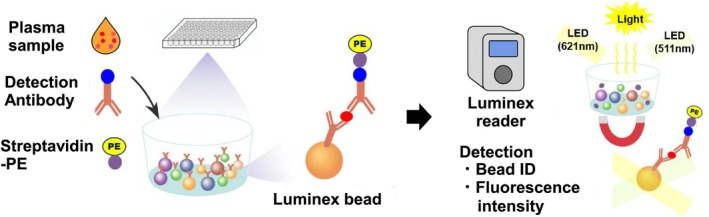
Principle of multiplex plasma biomarker measurement. Distinct fluorescently coded microspheres conjugated with analyte‐specific capture antibodies enable simultaneous detection of multiple plasma proteins within a single sample. After incubation with plasma samples, bound analytes are detected using biotinylated detection antibodies followed by streptavidin–phycoerythrin (PE). Signal intensity is quantified by fluorescence‐based detection with bead identity and reporter fluorescence measured by a Luminex reader and converted to absolute concentrations using analyte‐specific standard curves.

From an institutional cohort of 1195 patients who underwent surgical resection for CRC, right‐sided tumors were selected (Figure 2). After excluding left‐sided CRCs and cases without available preoperative plasma samples, 81 patients with paired tumor tissue and plasma samples were included for further analysis. Clinicopathological characteristics of the patients are summarized in Table 1. CDX2‐Low tumors were significantly associated with larger tumor size compared with CDX2‐High tumors (*p* = 0.018). In addition, CDX2‐Low tumors were more frequently associated with non‐differentiated histology (por/muc) than CDX2‐High tumors (*p* < 0.001). No significant differences were observed in age, sex, tumor stage, or metastatic status between the two groups (Table 1). CDX2 expression was assessed by immunohistochemistry on resected tumor specimens and classified using a standardized scoring system. Based on established criteria, tumors were categorized as CDX2‐Low (*n* = 15) or CDX2‐High (*n* = 66). Representative immunohistochemical images illustrating the scoring system are shown in Figure 2. Preoperative plasma samples from the same patients were subsequently analyzed using a multiplex ELISA platform to quantify circulating levels of selected cell surface markers, allowing direct comparison between tumor tissue expression and corresponding plasma concentrations (Figure 3). This approach minimizes sample volume requirements while preserving analytical sensitivity and dynamic range across multiple biomarkers. The clinicopathological characteristics observed in our cohort further support the biological aggressiveness of CDX2‐Low tumors. As shown in Table 1, CDX2‐Low tumors were associated with larger tumor size and a higher proportion of non‐differentiated histology, findings that are consistent with previous reports [4]. These features may reflect enhanced proliferative capacity and dedifferentiation associated with CDX2 loss.

**TABLE 1:** Patient characteristics.

Variable	CDX2‐High (*n* = 65*)	CDX2‐Low (*n* = 15)	*p*
Age, mean (SD)	72.2 (10.9)	73.5 (12.1)	0.679
T max (mm), mean (SD)	37.7 (21.6)	52.7 (22.4)	0.018*
Sex			0.128
F	31 (47.7%)	11 (73.3%)	
M	34 (52.3%)	4 (26.7%)	
Stage			0.388
0	6 (9.2%)	0 (0.0%)	
I	21 (32.3%)	2 (13.3%)	
II	20 (30.8%)	6 (40.0%)	
III	15 (23.1%)	7 (46.7%)	
IV	3 (4.6%)	0 (0.0%)	
Liver metastasis			0.229
+	4 (6.2%)	3 (20.0%)	
−	61 (93.8%)	12 (80.0%)	
Lung metastasis			0.324
+	2 (3.1%)	2 (13.3%)	
−	63 (96.9%)	13 (86.7%)	
Peritoneal metastasis			0.849
+	5 (7.7%)	2 (13.3%)	
−	60 (92.3%)	13 (86.7%)	
Pathology			< 0.001***
Differentiated (tub/pap)	63 (96.9%)	9 (60.0%)	
Others (por/muc)	2 (3.1%)	6 (40.0%)	

*Note:* One CDX2‐High patient was excluded from clinicopathological analysis due to missing data; T max (mm) represents a maximum tumor diameter.

* *p* < 0.05.

*** *p* < 0.001.

### 3.3 Circulating Levels of Tumor‐Associated Cell Surface Markers Do Not Reflect Tissue Expression

**FIGURE 4:**
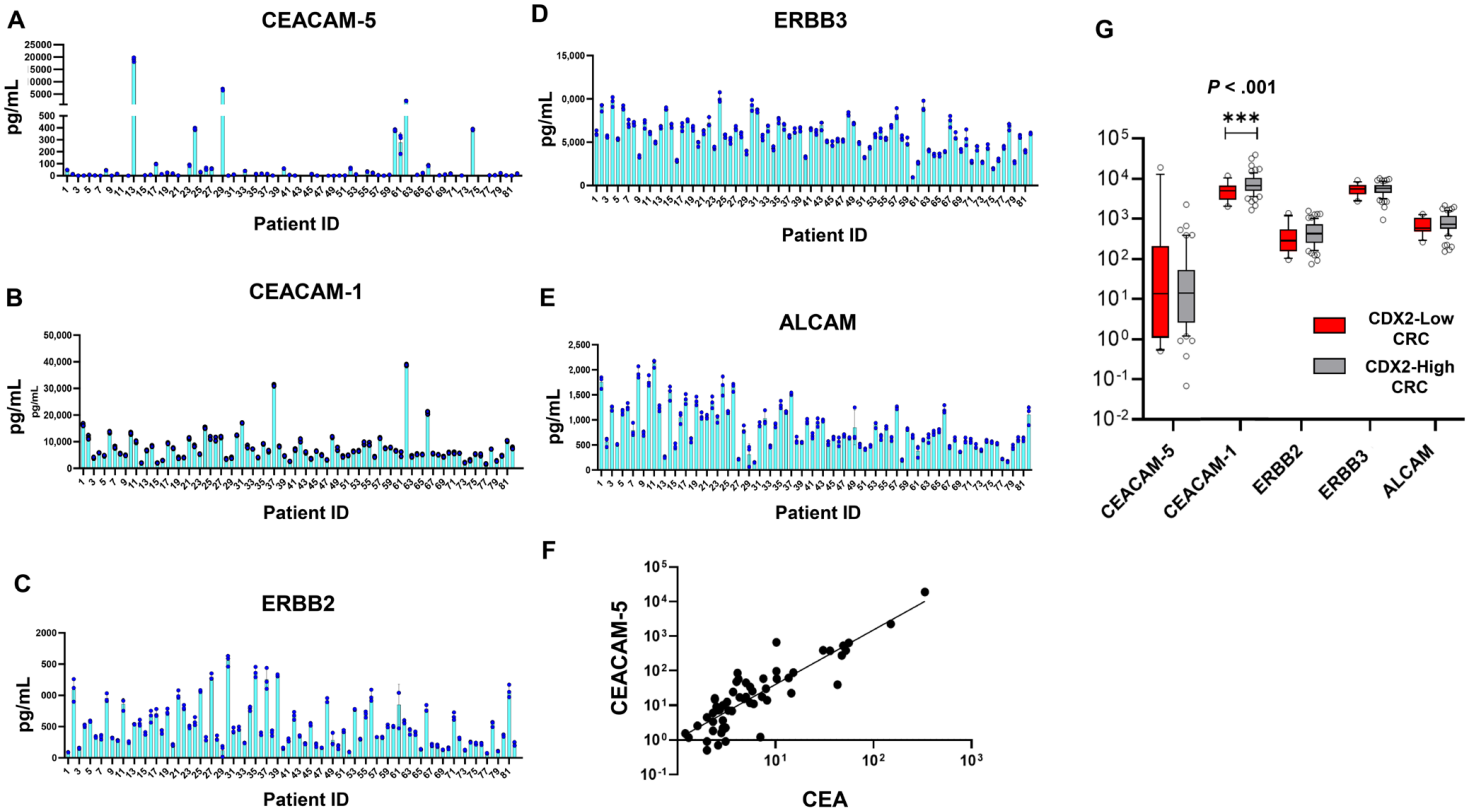
Plasma levels of tumor‐associated cell surface markers according to CDX2 status. (A–E) Per‐patient biomarker concentrations (pg/mL) measured in triplicate and summarized as bars (mean of triplicates): (A) CEACAM‐5 (CD66e), (B) CEACAM‐1 (CD66a), (C) ERBB2 (HER2), (D) ERBB3 (HER3), (E) ALCAM (CD166). (F) Correlation between CEA and CEACAM‐5 measured by Luminex (log10 scale) (line represents the linear regression line). (G) Boxplots showing the concentrations of the five biomarkers comparing CDX2‐Low (red) versus CDX2‐High (grey) determined by Human Luminex assays (Box‐and‐whisker plots show the median (line), interquartile range (box), and the 10th–90th percentiles (whiskers). Outliers are shown as individual points; two‐way ANOVA with Bonferroni's multiple comparisons test; ***p < 0.001).

Circulating concentrations of CEACAM5, ALCAM, ERBB2, and ERBB3 did not differ significantly between patients with CDX2‐Low and CDX2‐High CRC, despite the pronounced differences observed at the tissue level (Figure 4A–E). Individual patient‐level plots demonstrated substantial inter‐individual variability in plasma marker levels, with no consistent segregation according to CDX2 status.

To validate the reliability of the multiplex ELISA measurements, plasma CEACAM5 concentrations were compared with serum carcinoembryonic antigen (CEA) levels, a clinically established surrogate for circulating CEACAM5. A strong positive correlation was observed between CEACAM5 measured by multiplex ELISA and CEA values (Figure 4F), confirming the analytical validity of the assay.

Among the markers evaluated, CEACAM1 was the only analyte that exhibited a concordant pattern between tissue and plasma. Consistent with TCGA data, circulating CEACAM1 levels were significantly higher in patients with CDX2‐High tumors compared with those with CDX2‐Low tumors (*p* < 0.001; Figure 4G). No significant differences in tumor stage or nodal status were observed between the CDX2‐Low and CDX2‐High groups, suggesting that these clinicopathological factors had minimal influence on the circulating biomarker profiles.

## 4 Discussion

Our study suggests that tumor‐associated cell surface markers, even when highly overexpressed in the tumor, often lack utility as circulating biomarkers. This suggests that selecting biomarkers solely based on high tumor expression represents a suboptimal and potentially misleading strategy. In addition to post‐translational and systemic factors, this disconnect may also reflect disproportionate regulation at the translational level, whereby high mRNA expression does not necessarily translate into proportional protein production. Because tissue‐level differences were identified at the RNA level in TCGA, whereas circulating biomarkers reflect protein concentrations, RNA–protein discordance itself likely contributes to the observed translational gap. Together with biological barriers such as membrane retention, limited shedding, rapid degradation, or systemic clearance, these mechanisms can prevent tumor‐derived proteins from reaching detectable concentrations in circulation [5]. These findings highlight critical limitations in biomarker discovery: transcriptomic overexpression does not necessarily translate into blood detectability or functional relevance. However, because tissue transcriptomic data from TCGA and plasma protein data from ELISA‐based analyses were derived from independent cohorts rather than paired samples from the same patients, this gap should be interpreted cautiously. Future biomarker development should integrate expression profiling, secretion kinetics, release mechanisms, and validation of circulating presence using paired tissue and blood samples.

Approaches such as profiling tumor‐derived extracellular vesicles or circulating nucleic acids may yield more reliable blood‐based indicators [6, 7, 8]. Building on this concept, prior studies by our group and others indicate that circulating microRNAs may have potential as stable blood‐based markers, suggesting that nucleic acid–based approaches could complement protein‐based biomarker strategies [9, 10]. Other studies have suggested that expression levels of circulating microRNAs in plasma, as well as those of certain noncoding RNAs in tumor tissue, are associated with poor prognosis in CRC [11, 12, 13]. Rather than relying on a single biomarker, integrated panels comprising multiple noncoding RNAs or proteins may function as robust cancer detection biomarkers when coupled with machine learning‐based approaches [14], and may also serve as reliable predictors of recurrence [15]. There is also potential to utilize more readily measurable serum test items in clinical settings, such as cholinesterase, for predicting prognosis of CRC [16].

Although CEA, also known as CEACAM5, remains one of the serum biomarkers with established clinical utility in CRC, CEACAM1 emerged as a promising biomarker candidate in the present study because it showed concordance between TCGA‐based tissue transcriptomic analysis and ELISA‐based plasma analysis. CEACAM1 has been associated with recurrence and prognosis in patients with colorectal liver metastasis following hepatectomy [17, 18]. CEACAM1 expression levels have been reported to be downregulated in breast and prostate cancers, and CEACAM1 has been implicated in the regulation of tumor malignancy [19, 20]. In addition, CEACAM1 has been implicated in TIM‐3–mediated immune tolerance and exhaustion, and TIM‐3/CEACAM1 co‐expression has been associated with T‐cell exhaustion in patients with CRC [21, 22]. Taken together, these reports support the biological and clinical relevance of CEACAM1 and are consistent with our findings, which demonstrate lower CEACAM1 levels in both CDX2‐Low tumor tissue and plasma from CDX2‐Low cases compared with CDX2‐High cases. CEACAM1 expression can be silenced by interferon regulatory factor 1 (IRF1) and by a variant of heterogeneous nuclear ribonucleoprotein L (hnRNP L) via an alternative splicing mechanism [23, 24]. Thus, targeting splicing factors that suppress CEACAM1 expression holds potential as a novel therapeutic target for CRC. However, CEACAM1 should be regarded as an exploratory biomarker candidate in the present study, as diagnostic performance analyses and survival validation were not performed. Moreover, because tissue and plasma data were not obtained as paired samples from the same patients, the concordance observed for CEACAM1 should be interpreted as hypothesis‐generating. Prospective studies are required before clinical implementation can be considered.

Importantly, CDX2‐Low CRCs represented a biologically aggressive subtype associated with poor differentiation and unfavorable prognosis. However, CDX2 immunohistochemical evaluation is not routinely implemented in all clinical settings. Therefore, identification of circulating biomarkers capable of noninvasively identifying CDX2‐Low tumors would have substantial clinical value for risk stratification and treatment decision‐making. This concept is also consistent with the increasing recognition that biomarker development in gastrointestinal cancers should move beyond single genomic indices and incorporate biologically meaningful subtype and immune‐related contexts [25]. In this context, CDX2‐based stratification was not used merely as an analytical framework but as a clinically motivated model to test whether biologically meaningful subtype differences are reflected in circulation.

This study has several limitations. The number of CDX2‐Low cases was relatively small (*n* = 15), which may have limited statistical power to detect subtle differences in circulating biomarker levels. Although the absence of concordance was consistent across multiple analytes, larger validation cohorts are required to confirm these findings. Comprehensive molecular profiling data (including *KRAS*, *NRAS*, and *BRAF* mutations and MSI status) were not uniformly available in this cohort. As molecular alterations may influence tumor biology and circulating protein levels, the absence of systematic molecular stratification represents a limitation of the present study. The present cohort was limited to right‐sided CRC, given the known association between CDX2 loss and proximal tumor biology. Right‐ and left‐sided CRC differ not only anatomically but also in mutation profiles, immune‐related features, and consensus molecular subtype distribution, which may influence both tumor marker expression and circulating biomarker detectability [26, 27]. Therefore, the biomarker profiles and tissue‐to‐plasma relationships observed in this study may not be fully generalizable to left‐sided CRC or rectal cancer. Whether similar translational gaps exist in left‐sided CRC remains uncertain and warrants further investigation. Future investigations integrating genomic, transcriptomic, and circulating proteomic analyses within the same cohort will be necessary to further refine the development of subtype‐specific biomarkers.

Therefore, continued efforts are needed to identify and validate novel biomarker candidates derived from experimental studies and clinical observations, such as those presented in our study. Overall, our findings underscore the need to bridge tissue‐level discovery and systemic biology to translate biomarkers into clinical practice.

## Author Contributions

**Shigenori Suzuki:** data curation, resources. **Sang‐Woong Lee:** supervision. **Hiroki Hamamoto:** data curation, resources. **Dai Shida:** supervision. **Junko Mukohyama:** conceptualization, investigation, methodology, formal analysis, funding acquisition, writing – original draft, writing – review and editing, software, project administration, visualization, validation. **Kohei Taniguchi:** data curation, formal analysis, writing – original draft, resources. **Xinyi Fu:** writing – review and editing, formal analysis. **Kimihiro Yamashita:** writing – review and editing, supervision, formal analysis. **Akihide Yoshimi:** supervision, formal analysis, investigation, writing – review and editing, writing – original draft, visualization, validation, methodology. **Shohei F. Fujita:** writing – review and editing, formal analysis, resources.

## Funding

Junko Mukohyama was partly supported by funding from the Japan Society for the Promotion of Science (JSPS) Grant‐in‐Aid for Scientific Research (C) (grant number 24K11942); Lotte Foundation; Kobayashi Foundation; Kato Memorial Bioscience Foundation; The Naito Foundation; Astellas Foundation for Research on Metabolic Disorders; The Yasuda Medical Foundation; Japan Surgical Society (JSS) Young Researcher Award. Kohei Taniguchi was partly supported by the JSPS Grants‐in‐Aid for Scientific Research (B) (25K02711); Research Support Project for Life Science and Drug Discovery (Basis for Supporting Innovative Drug Discovery and Life Science Research (BINDS)) from AMED (grant numbers JP25ama121052 and JP25ama121054). Akihide Yoshimi was partly supported by the JSPS Grants‐in‐Aid for Scientific Research (A) (grant number 21H04828); the Research on Development of New Drugs from the Japan Agency for Medical Research and Development (AMED) (project number JP25ak0101263); the Practical Research for Innovative Cancer Control from AMED (project number JP25ck0106946; JP25ck0106906); grants from Princess Takamatsu Cancer Research Fund and Takeda Science Foundation.

## Ethics Statement

This study was conducted in accordance with the Declaration of Helsinki and was approved by the Ethics Committees of all participating institutions. Ethical approval was obtained from the Institute of Medical Science, The University of Tokyo (approval no. 2024‐21‐0809), the National Cancer Center (approval no. 2021‐361 and 2020‐486), and Osaka Medical and Pharmaceutical University (approval no. 2305‐15 and 2344‐15).

## Conflicts of Interest

J.M. reports receiving consulting fees from Nippon Kayaku Co. Ltd., outside the submitted work. A.Y. received research funding from Daiichi Sankyo, Chugai Pharmaceutical, and Eisai unrelated to this study; and received honoraria from Oxford Nanopore Technologies, Chordia Therapeutics, Otsuka Pharmaceutical, MSD, Kyowa Kirin, Daiichi‐Sankyo, Sanofi, Sumitomo Dainippon Pharma, Life Technologies Japan, and Amgen. Junko Mukohyama was partly supported by funding from the Japan Society for the Promotion of Science (JSPS) Grant‐in‐Aid for Scientific Research (C) (grant number 24 K11942); Lotte Foundation; Kobayashi Foundation; Kato Memorial Bioscience Foundation; The Naito Foundation; Astellas Foundation for Research on Metabolic Disorders; The Yasuda Medical Foundation; Japan Surgical Society (JSS) Young Researcher Award. Kohei Taniguchi was partly supported by the JSPS Grants‐in‐Aid for Scientific Research (B) (25 K02711); Research Support Project for Life Science and Drug Discovery (Basis for Supporting Innovative Drug Discovery and Life Science Research (BINDS)) from AMED (grant numbers JP25ama121052 and JP25ama121054). A.Y. was partly supported by the JSPS Grants‐in‐Aid for Scientific Research (A) (grant number 21H04828); the Research on Development of New Drugs from the Japan Agency for Medical Research and Development (AMED) (project number JP25ak0101263); the Practical Research for Innovative Cancer Control from AMED (project number JP25ck0106946; JP25ck0106906); grants from Princess Takamatsu Cancer Research Fund and Takeda Science Foundation.

## Supporting information


**Table S1:** Circulating marker levels according to CDX2 expression status.

## Data Availability

The data used from The Cancer Genome Atlas (TCGA) are publicly available (https://portal.gdc.cancer.gov/). Clinical data from our institutional cohort are not publicly available to protect patient privacy; however, ELISA data generated for this study are available from the corresponding author upon reasonable request.
