# Supplementary material for: Translational Gap in Biomarker Discovery: Tumor Surface Markers Rarely Mirror Circulating Levels
Source: Ann Gastroenterol Surg. 2026 Jun 5:10.1002/ags3.70240. Online ahead of print. doi: 10.1002/ags3.70240 (PMC13394966; doi:10.1002/ags3.70240)
Supplement: Supplementary file 1 — Table S1: Circulating marker levels according to CDX2 expression status. [file AGS3-9999-0-s001.pdf]

Supplementary Table 1. Circulating marker levels according to CDX2 expression status

Marker	CDX2-High, median (IQR)	CDX2-Low, median (IQR)
**CEACAM5**	14.65 (2.71–49.01)	13.84 (1.55–58.03)
**CEACAM1**	6706.99 (4864.85–10175.36)	5036.07 (2947.49–6578.00)
**ErbB2**	441.21 (258.65–734.55)	264.76 (158.29–533.28)
**ErbB3**	5830.19 (4402.88–6872.13)	5397.53 (4144.36–7078.95)
**ALCAM**	710.49 (543.38–1151.90)	624.34 (463.05–1096.08)

Data are presented as median (IQR). IQR, interquartile range (25th–75th percentile).

Difference (CDX2-Low － CDX2-High)	95% CI
1713.11	58.36 to 3367.85
-2969.32	-4125.15 to -1813.48
-108.42	-228.70 to 11.86
-158.31	-759.15 to 442.53
-121.65	-244.80 to 1.49
